# Transplantation of selected or transgenic blood stem cells – a future treatment for HIV/AIDS?

**DOI:** 10.1186/1758-2652-12-10

**Published:** 2009-06-28

**Authors:** Gero Hütter, Thomas Schneider, Eckhard Thiel

**Affiliations:** 1 Medical Department III (Hematology, Oncology), Charité Universitätsmedizin Berlin, Campus Benjamin Franklin, Berlin, Germany; 2 Medical Department I (Gastroenterology, Infectious Diseases and Rheumatology), Charité Universitätsmedizin Berlin, Campus Benjamin Franklin, Berlin, Germany

## Abstract

Interaction with the chemokine receptor, CCR5, is a necessary precondition for maintaining HIV-1 infection. Individuals with the CCR5-delta32 deletion who lack this receptor are highly resistant to infection by the most common forms of HIV-1. We recently reported on the successful transplantation in an HIV-1-positive patient of allogeneic stem cells homozygous for the CCR5-delta32 allele, which stopped viral replication for more than 27 months without antiretroviral therapy.

Here, we report on the results of a meeting regarding the potential implications and future directions of stem cell-targeted HIV treatments. The meeting drew together an international panel of hematologists, immunologists, HIV specialists and representatives from bone marrow donor registries.

The meeting came to an agreement to support further attempts to use CCR5-delta32 deleted stem cells, for example, prescreened cord blood stem cells, to treat probable HIV-1-positive patients with malignancies. Furthermore, improvement of HIV-1 therapy that interferes with the entry mechanism seems to be a promising approach in HIV-1-infected patients with no matching CCR5-delta32 deleted donor.

## Introduction

Entry of the HIV-1 into the host cells requires the interaction of the viral envelope with the CD4 surface molecule and certain co-receptors, predominantly represented by the chemokine receptor, CCR5. Blocking the co-receptor interaction of CCR5-tropic HIV-1 by small-molecule antagonists proved to be highly efficacious, suppressing HIV-1 replication in extensively pretreated patients with multi-resistance and virological failure of preceding regimens [1].

Previously, a 32 base pair deletion in the CCR5 gene (CCR5-delta32), leading to a truncated gene product, had been shown to confer marked protection against HIV-1 infection in homozygous individuals, while infected heterozygotes show substantially delayed progression of the infection [2,3].

### First case of long-term HIV control by stem cell transplantation

In the 12 February 2009 issue of the *New England Journal of Medicine*, we reported on an HIV patient with acute myeloid leukemia who achieved long-term control of HIV-1 after allogeneic hematopoietic stem cell transplantation (alloHSCT) from a human leukocyte antigen (HLA) matched unrelated donor homozygous for CCR5-delta32 [4]. The patient was classified as being in CDC Stage 2, and had been on HAART for five years with a proportion of CXCR4 using strains (×4) of 2.9% before transplantation.

Viral load remained below the limit of detection 27 months after transplantation, despite discontinuation of antiretroviral therapy. This result underscores the essential role of the CCR5 co-receptor in maintaining HIV replication and raises questions about the feasibility of HIV eradication by stem cell transplantation-based approaches.

### Expert panel discussed future directions

On 20 April 2009, an international panel of hematologists, HIV specialists and representatives from bone marrow donor registries (ZKRD Ulm, Germany, and BBMR, Bristol and London, UK) and donor centres (DKMS, Tübingen, Germany, and Stefan-Morsch-Stiftung, Birkenfeld, Germany) met at a Berlin venue to discuss potential implications and potential future directions of research that emerge from this breakthrough observation. The workshop was encouraged by Malcolm Thomas, trustee of the British Bone Marrow Donor Appeal, and was chaired by E Thiel from the Medical Department III of the Charité University Hospital, Berlin, Germany.

Combination antiretroviral therapy (ART) allows for long-term suppression of HIV-1 replication below the level of detection in the majority of patients, thus greatly reducing the percentage of patients progressing to AIDS. Life expectancy in HIV-infected patients treated with HAART has increased in the past 10 years, although there is considerable variability between subgroups of patients. In high-income countries, the average life expectancy at the age of 20 years for HIV-positive people receiving ART is about two-thirds of that of the general population [5].

However, this success has required the development of more than 20 antiretroviral drugs since the first isolation of the virus 25 years ago, and a substantial number of patients still end up with multi-resistant viruses and very limited therapeutic options. As M Bickel (JW Goethe University, Frankfurt/M, Germany) emphasized in his talk, drug resistance, side effects, comorbidity and adherence now emerge as the main factors that limit treatment efficacy.

Furthermore, it has been suggested that the maintenance of viral reservoirs, for example, in gut-associated lymphoid tissues, play a major role in the persistence of HIV [6]. Even today, patients are dying from HIV infection or HIV-related diseases despite state-of-the-art antiretroviral therapy. Long-term outcomes may be improved by starting ART earlier, i.e., when CD4+ T cell count levels are higher, but permanent abrogation of virus replication will remain a medical need unmet by conventional therapeutic approaches.

Since ART treatment costs in the range of €30,000 per year, definitive therapies abolishing the need for life-long antiviral treatment should be beneficial even in terms of utilization of health-care resources.

### Therapy with CCR5-negative stem cells

In the early 1980s, alloHSCT appeared to be attractive as a therapy for HIV in patients with advanced disease because it was thought to substitute depleted CD4 cells and reduce the HIV reservoir via the conditioning myeloablative therapy.

As G Hütter (Charité Berlin, Germany) pointed out in his talk, previous attempts of alloHSCT in HIV patients with hematological malignancies produced encouraging overall survival outcomes, but these therapies failed to provide a benefit in terms of HIV viral load reduction without continued ART. At least, HIV infection did not progress despite immunosuppression, and alloHSCT appears medically feasible in HIV patients with ongoing ART [7].

The case reported by Hütter et al now provides a proof of principle for CCR5-targeted stem cell therapies. CCR5-delta32 status appears to have several beneficial effects on the alloHSCT setting: previous analyses revealed that the CCR5-delta32 allele appears to protect against acute graft versus host disease (GVHD) and EBV reactivation [8,9].

In contrast, the consequences of transplanting CCR5-negative donor cells to CCR5-positive recipients have so far not been fully elucidated. For example, the reduction of GVHD in the CCR5-delta32 setting raises the question of whether CCR5 negativity may be associated with a diminished graft versus leukemia effect. However, the patient described by Hütter et al developed a GVHD, which is somewhat reassuring in terms of the reactivity of CCR5-negative lymphocytes. Still, the sudden acquired lack of CCR5, in contrast to life-long absence of the chemokine receptor, may have yet unknown detrimental effects, which are not observed in hereditary CCR5-negative individuals because of compensatory adaptations of the cytokine receptor network.

In the gut mucosa of the reported patient, CCR5-positive macrophages were detected five months after transplantation. Although complete chimerism of the myeloid lineage had not been reached at this time, viral rebound was not observed. Mucosal macrophages are known to serve as long-term virus reservoirs, as T Schneider (Charité Berlin, Germany) pointed out in his presentation. The case illustrates that eradication of the primary target cells may be sufficient to prevent a rebound of viral replication form these reservoirs.

Genotypic determination of co-receptor tropism by ultra-deep sequencing before alloHSCT had revealed that the patient harboured a minor fraction of X4 viruses, which might have been expected to take over after elimination of CCR5-positive lymphocytes. However, neither CCR5-using nor CCR4-using variants have been detected in the follow ups so far.

This raises the possibility that CCR5-delta32 expression has a dominant negative effect on CXCR4-mediated viral entry [10]. In addition, the shift to X4 variants seen in some patients with long-standing infections may be a gradual process in which CCR5-using viruses somehow pave the way for X4-using variants, a process that may have been prevented by the sudden withdrawal of the target cells.

### Stem cell sources

The CCR5-delta32 allele is mostly limited to the caucasian population, with the highest frequency being reached in the north-eastern parts of Europe [11]. It is largely absent in Africa, as well as in eastern and south-eastern Asia. Prevalence of the homozygous carriers is in the range of 1% to 3% among caucasians. Future approaches to HIV therapy by CCR5-negative alloHSCT may thus be limited by the availability of HLA-matched donors in general and in the non-caucasoid populations in particular.

C Müller illustrated this fact with data from the German National Bone Marrow Donor Registry (ZKRD, Ulm, Germany): a simulation study based on high-resolution HLA-A, B and DRB1 haplotype frequencies reveals that with the current registry size, about 75% of German patient will find at least one allele-matching donor for these loci. Assuming a prevalence of 3% CCR5-delta32 homozygotes, the likelihood of finding a matched German donor is reduced to 30% if donors carrying two CCR5-delta32 alleles are sought. Bringing this figure back up to 75% would require an expansion or the donor pool by a factor of at least 10.

However, this problem is alleviated by the worldwide cooperation of stem cell registries in the Bone Marrow Donors Worldwide and European Marrow Donor Information System networks, which are currently in the process of merging into one single global registry access system. Biostatistics will become more and more effective in using limited HLA typing information to narrow down the set of potential donors for a given patient, so these individuals could be tested for CCR5-delta32 before costly confirmatory HLA typing is undertaken.

In an effort to facilitate CCR5-targeted stem cell therapy, Chow et al established a cord blood bank with specimens from 10,000 CCR5-screened donors [12]. However, this source of stem cells has not been used for transplantation in HIV patients yet. Cord blood may emerge as an important source of CCR5-negative stem cells because they have been used successfully, even in the situation of a partial donor-recipient mismatch. However, this approach may be complicated by the fact that cord stem cell transplantation in adults currently requires more than one cord blood unit.

### Donor recruitment

A major issue concerning the supply of CCR5-negative stem cells is the donor information policy of the bone marrow registries. Transplantation from units of umbilical cord blood stem cell only requires 4/6 HLA matches at HLA A, B and DR with Class I matches, which would remarkably increase the probability of finding a matching donor with CCR5-delta32 homozygosity.

C Navarrete (British Bone Marrow Registry, London, UK) explained that in cord blood banks in particular, testing of CCR5 status is mostly not covered by the informed consent signed by mothers. Thus at present, it is not legally acceptable to screen existing units post hoc for CCR5-negativity. The same applies to adult donor recruitment programmes: CCR5 status testing may not be currently covered by the informed consent given by volunteers. Information strategies must therefore be carefully devised in order to avoid detrimental effects to volunteer-unrelated donor recruitment.

### Using HIV resistance transgenes in stem cells

Another approach to the provision of HIV-resistant blood cells is the introduction of resistance-conferring genes into stem cells before transplantation. This is an attractive option: it may be a once-in-a-lifetime treatment; it is expected to obviate or greatly reduce the need of ART; and it may be suitable for patients with restricted ART options due to resistance or side effects.

Since it is not possible to achieve gene transfer into all cells of a transplant, the therapeutic gene must confer a selective advantage allowing for the expansion of the transgenic cell pool in vivo, which then will then gradually replace virus-susceptible cells. This is best reached by targeting steps in the viral life cycle that protect the transgenic cells from the cytopathic effects of viral components produced inside the cell.

However, gene therapy approaches using so-called Class II target genes, i.e., those that inhibit the synthesis of virus particles but still allow for proviral integration of infecting virus, have largely been unsuccessful. Thus, targeting steps before integration (Class I target genes), and inhibition of entry in particular, is the most promising approach, a notion of the long-term fate of hematopoietic cells carrying transgenes directed against different steps in the viral life cycle [13].

B Fehse (Department of Cellular and Gene Therapy Research, Hamburg-Eppendorf, Germany) presented an ongoing Phase I/II gene therapy trial in HIV-positive patients. The transgene used in this study encodes a membrane-anchored peptide (C46) that interferes with the viral-cellular membrane fusion, mechanistically similar to the fusion inhibitor enfuvirtide. A Phase I study using transgenic T cells without conditioning regimen had shown good tolerability and long-term survival, over one year of follow up, of marked cells in some patients [14].

The ongoing Phase I/II will use C46-transfected autologous stem cells in up to 10 patients with an independent indication for autoHSCT, i.e., high-risk AIDS-related lymphoma. Use of autologous stem cells for gene transfer currently requires a biosafety reduced S3 laboratory environment for the transgene introduction. Future gene therapy approaches may involve allogeneic transplants, obviating the need for laboratory conditions of high safety levels, and exploiting the graft-versus-neoplasm effect in patients with malignancies.

Recruitment of patients into this type of studies should be facilitated by the nationwide and international activities of the German Competence Network HIV/AIDS, represented by K Jansen (Bochum, Germany) at this meeting.

## Conclusion

CCR5-negative stem cell transplantation and transgenic approaches to HIV therapy hold great promise for future curative interventions in HIV patients. Replacing lifelong HAART by a once-in-a-lifetime treatment would have numerous benefits for patients and the health care system.

If the results published by Hütter et al could be reproduced in a few additional HIV-infected leukemia patients, and the legal questions associated with the use of registry donors are resolved, the way would be open for intensified and pre-emptive CCR5-delta32 donor screening for patients with HIV and malignant diseases.

Similar considerations apply to the described gene therapeutic approach, which may be adopted more widely due to the opportunity to use autologous stem cells. Further experiences with CCR5-targeted stem cell therapies will probably encourage the treatment of selected populations of young HIV-positive patients with multi-resistant infection and exhaustion of CD4 cells, as well as HIV-infected pediatric patients with rapidly progressing disease.

## Competing interests

The authors declare that they have no competing interests.

## Authors' contributions

GH organized the meeting and wrote the manuscript. ET and TS wrote the manuscript. All authors read and approved the final manuscript.
